# Acute Effects of Dermal Suction on Passive Muscle and Joint Stiffness

**DOI:** 10.3390/healthcare9111483

**Published:** 2021-10-31

**Authors:** Shota Enomoto, Tomonari Shibutani, Yu Akihara, Miyuki Nakatani, Kazunori Yamada, Toshiaki Oda

**Affiliations:** 1 Center for Liberal Arts, Meiji Gakuin University, Yokohama 244-8539, Japan; mnaka@gen.meijigakuin.ac.jp; 2 Institute of Sports Sciences, International Pacific University, Okayama 709-0863, Japan; t-shibu@mj-company.co.jp; 3 MJ Company. K.K., Okayama 700-0953, Japan; 4 Fourleaf. K.K., Okayama 700-0953, Japan; 5 Graduate School of Education, Hyogo University of Teacher Education, Kato 673-1494, Japan; ijirinisi@yahoo.co.jp (Y.A.), toda@hyogo-u.ac.jp (T.O.); 6 Department of Community Child Studies, Niijimagakuen Junior College, Takasaki 370-0068, Japan; kazu-yamada@mail.neesima.ac.jp

**Keywords:** elastography, flexibility, medial gastrocnemius, shear wave velocity, ultrasound

## Abstract

The aim of the present study was to examine the acute effects of dermal suction on the passive mechanical properties of specific muscles and joints. Dermal suction was applied to the calves of 24 subjects. Passive plantar flexion torque was measured with the right knee fully extended and the right ankle positioned at 20°, 10°, 0°, and −10° angles, where 0° represents the ankle neutral position, and positive values correspond to the plantar flexion angle. The shear wave velocity (SWV) (m/s) of the medial gastrocnemius was measured in the same position using ultrasound shear wave elastography. The relationship between the joint angle and passive torque at each 10° angle was defined as passive joint stiffness (Nm/°). Passive muscle and joint stiffness were measured immediately before and after the dermal suction protocol. When the ankle joint was positioned at 20° (*r* = 0.53, *P* = 0.006), 10° (*r* = 0.43, *P* = 0.030), and −10° (*r* = 0.60, *P* = 0.001), the SWV was significantly higher after dermal suction than that before dermal suction. Regarding joint stiffness, we found no significant difference between the pre- and post-dermal suction values (*partial η*^2^ = 0.093, *P* > 0.05). These findings suggest that dermal suction increases passive muscle stiffness and has a limited impact on passive joint stiffness.

## 1. Introduction

Flexibility is a component of physical fitness, and various studies have been conducted to research ways to improve flexibility [1]. There are many methods of improving flexibility, dermal suction being one of them. In a traditional Chinese medicine-based therapy, dermal suction is known as cupping therapy. Cupping therapy is a treatment method that involves application of a vacuum to a localized area of the skin [2]. The mechanism of action of cupping therapy is not clear [3]; however, cupping has been reported to be beneficial for pain, herpes zoster, and other diseases [4]. Regarding flexibility, some studies have reported that cupping therapy increases the joint range of motion (ROM) [5,6,7,8], whereas another study demonstrated no significant change in the joint ROM after cupping therapy [9].

As above-mentioned, previous studies evaluated the influence of cupping therapy on joint flexibility using joint ROM as an index of joint flexibility [5,6,7,8,9]. However, all the structures located around the joint including the muscles, tendons, skin, subcutaneous tissue, joint capsule, and cartilage, contribute to joint flexibility [10]. In previous experiments involving only joint flexibility [5,6,7,8,9], it was unclear what the changes in the joint ROM can be attributed.

Ultrasound elastography is a technique that can localize stiffness or hardness of a muscle [11]. Muscle stiffness has been defined as the ratio of change in force to change in length along the long axis of a muscle (proximal to distal direction of the muscle belly) [12], whereas muscle hardness has been defined as the resistance offered by the muscle against perpendicular pressure [13]. Several different ultrasound elastography methods including shear wave elastography and strain elastography are currently used [11]. Each of them uses a different physical principle to measure the mechanical properties of the target tissue [14]. Shear wave elastography and strain elastography assess the muscle stiffness and muscle hardness, respectively [11].

Recently, to examine whether cupping therapy could change mechanical properties of muscle, Jan et al. [15] investigated the effect of cupping therapy on muscle hardness in the triceps of 12 healthy subjects using strain elastography and reported that cupping therapy significantly reduces muscle hardness. However, the ROM of a joint is considered to be influenced by the longitudinal mechanical property of a muscle (i.e., muscle stiffness). Thus, in the context of the mechanism underlying the improvement of joint flexibility caused by dermal suction, muscle stiffness needs to be examined. Elucidation of the dermal suction effects on passive muscle stiffness using shear wave elastography may aid in our understanding of the mechanisms of improvement in joint flexibility achieved using dermal suction, and provide useful information for physical therapists and conditioning professionals who use dermal suction on muscles or joints in their clinical practice. The aim of our study, therefore, was to examine the acute effects of dermal suction on the passive mechanical properties of specific muscles and joints to verify the hypothesis that dermal suction decreases passive muscle stiffness and improves joint flexibility.

## 2. Materials and Methods

### 2.1. Subjects

We enrolled 12 men and 12 women who were recreationally active in this study. None of the subjects reported any ongoing neuromuscular diseases or musculoskeletal injuries specific to the ankle joint. In addition, no subject complained about poor physical condition on the measurement day. The age and physical characteristics of the subjects are shown in Table 1. Before the survey, we explained the purpose, content, methods, and risks of the study to the subjects. We obtained written informed consent from each participant. The present study was approved by the local ethics committee.

SD: standard deviation, CI: confidence interval, lb: lower bound, ub: upper bound, BMI: body mass index.

### 2.2. Procedure

This study was a single-arm study designed to examine the acute effects of dermal suction on passive muscle and joint stiffness. After measuring ankle joint stiffness using a dynamometer and the passive stiffness of the medial head of the gastrocnemius of the right leg using ultrasound shear wave elastography, dermal suction was applied to the right calf of the subjects for 8 min, and the same measurements were taken immediately after the application.

### 2.3. Passive Ankle Joint Stiffness

**Figure 1:**
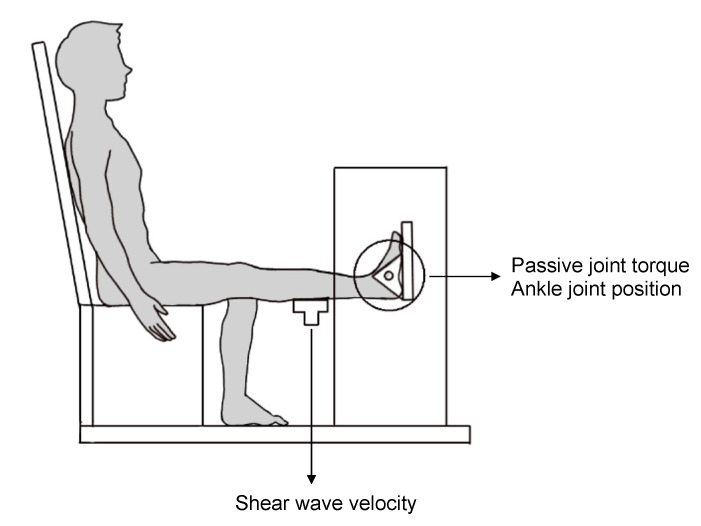
Experimental setup. Passive plantar flexion torque of the ankle joint and the shear wave velocity (SWV) (m/s) of the medial gastrocnemius were measured using a dynamometer and ultrasound shear wave elastography, respectively.

Passive plantar flexion torque was measured using a specially designed dynamometer (Vine, Tokyo, Japan), which has previously been used by other investigators to measure the plantar flexion torque [16,17,18]. The subjects were seated on the bench of the dynamometer with the knee fully extended while the right ankle was set on the footplate and fastened and restricted to relax the muscles at each joint position (Figure 1). The footplate was fixed with the ankle positioned at 20°, 10°, 0° (neutral position), and −10°. The positive and negative values corresponded to the plantar flexion and dorsiflexion angles, respectively. The footplate was passively and manually dorsiflexed at a low speed. The ankle was returned to the 20° position before testing the next position. For testing, each joint position was maintained for approximately 10 s. The torque data were imported into a personal computer at a sampling frequency of 1000 Hz using an A/D converter (PowerLab 16/35, AD Instruments, Australia). The relationship between the joint angle and passive torque at each 10° was defined as passive joint stiffness. Four training plates of different weights were used to investigate the reliability and validity of the dynamometer. Loads of 12.25 Nm, 24.5 Nm, 36.75 Nm, and 49 Nm were applied, and each load was measured 10 times. The coefficient of variations (CV), standard error of the mean (SEM), and intraclass correlation coefficients (ICC) were calculated. Pearson’s correlation coefficient was used to test the significance of the relationship between torque values measured by the dynamometer and the loads. Torque values measured by the dynamometer were 12.27 (0.31) Nm, 24.83 (0.38) Nm, 36.85 (0.28) Nm, and 49.01 (0.14) Nm. CV for loads of 12.25 Nm, 24.5 Nm, 36.75 Nm, and 49 Nm were 2.54%, 1.53%, 0.76%, and 0.28%, respectively. The SEM for four loads were 0.10, 0.12, 0.09, 0.04 (Nm), respectively. ICC (1,1) was 1.000 [95% confidence interval, 0.999 to 1.000]. The correlation coefficient between torque values measured by the dynamometer and the loads was 0.999.

### 2.4. Passive Muscle Stiffness

During the measurement of passive ankle joint stiffness, passive muscle stiffness was simultaneously measured in the same position (Figure 1). The passive muscle stiffness of the medial gastrocnemius (MG) was measured using an ultrasound shear wave elastography scanner Aixplorer (Supersonic Imagine, Aix-en-Provence, France) with a linear array transducer SL15-4 (Supersonic Imagine, Aix-en-Provence, France). The validity of shear wave elastography has been confirmed using phantoms [19] and experimental animals [20]; moreover, the measurement of human muscle stiffness by shear wave elastography has been previously reported [21]. In the present study, shear wave velocity (SWV) (m/s) was used as an index of muscle stiffness. The probe was placed on the MG at 30% of the lower leg length (the distance between the popliteal crease and the center of the lateral malleolus). The SWV was calculated over the largest region of interest from which we excluded the aponeurosis and subcutaneous adipose tissue. SWV analysis was performed using the software (Q-Box) built into the ultrasound shear wave elastography scanner. The average of three images at each joint angle was calculated and used for the analysis. To synchronize the SWV and other data, the computer and ultrasonic diagnostic equipment times were synchronized. We investigated the reliability using eight subjects. The stiffness of MG was measured six times for each subject while keeping the subject seated on the bench of the dynamometer and fixing the footplate with the ankle positioned at 20° of plantar flexion. The mean values of CV and SEM for eight subjects were 4.43% and 0.05 (m/s). ICC (1, 1) was 0.767 [95% confidence interval, 0.535 to 0.936].

### 2.5. Dermal Suction

**Figure 2:**
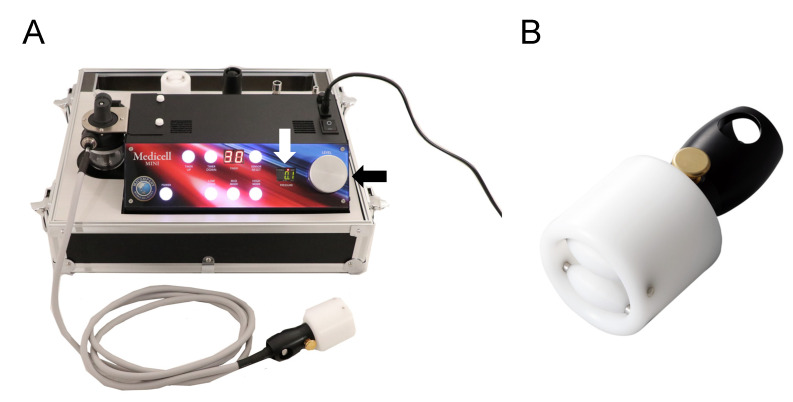
The instrument used in dermal suction. Medicell consists of a part that generates negative pressure (**A**) and a cup that is used to apply suction onto the skin (**B**). The negative pressure is displayed on the screen (white arrow) in real time and can be adjusted using a dial (black arrow). To improve the sliding of the cup during dermal suction, a roller is present in the cup (**B**).

**Figure 3:**
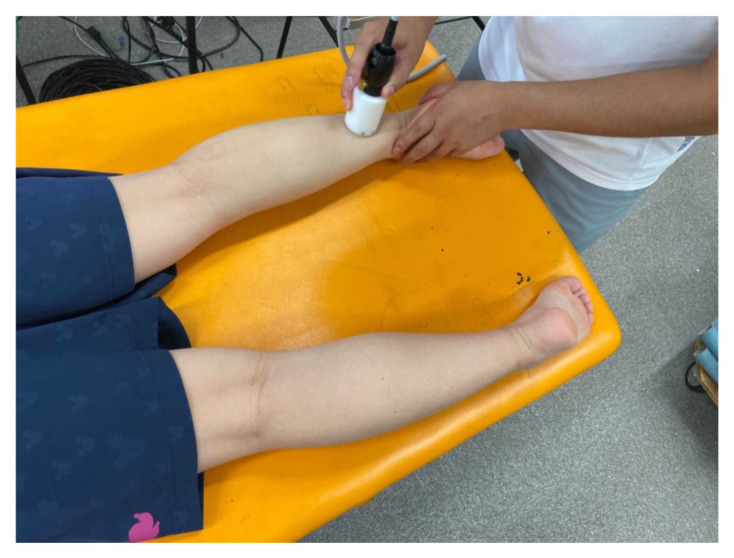
Illustration of the process of dermal suction. Dermal suction applied to the calf muscles.

Dermal suction was performed using Medicell (MJ Company. K.K., Okayama, Japan) (Figure 2), which consists of a part that generates negative pressure and a cup that applies suction onto the skin. The negative pressure is displayed on the screen in real time. The internal and external diameters of the cup were 2.96 cm and 4.60 cm, respectively. The cup sizes in this study were similar to those of cups typically used in cupping treatment, which have diameters in the range of approximately 3.80 cm to 5.08 cm [22]. To improve the sliding of the cup during dermal suction, a roller is present in the cup. Dermal suction using Medicell was performed for a total of 8 min (4 min initially, followed by a 30-s rest period, and then 4 min more) on the right calf (Figure 3). The total treatment time was maintained similar to that in the study by Yim et al. [8]. In our preliminary experiments, treating a dermal suction in a constant rhythm for 8 min was deemed as a technical difficulty. Hence, a rest period was provided. During the treatment, while lying prone on a massage table, the subjects were instructed to relax the muscles. Dermal suction targeted the calf muscles, and the cup was slid between the origin and insertion of the gastrocnemius at approximately 0.5 Hz (Figure 3). The intensity of dermal suction was 20 kPa of negative pressure and was adjusted before each treatment. In cupping therapy, the negative pressure is recommended to be between 225 and 300 mmHg (i.e., 30 to 40 kPa) [15]. In contrast, in our preliminary experiments, some subjects complained of strong pain when dermal suction was performed at this intensity (30 to 40 kPa) using the equipment of this study. Therefore, an intensity of 20 kPa was selected. A baby oil (Johnson & Johnson K. K., Tokyo, Japan) consisting of mineral oil and tocopherol acetate was applied to the skin to improve the sliding of the cup.

### 2.6. Statistics

Statistical analyses were performed using SPSS statistics (Version 26, IBM Corporation, Armonk, New York). In order to verify whether there was a difference in the SWV and the joint stiffness due to dermal suction and changes in the joint angles, a two-way repeated measures analysis of variance (ANOVA) was performed, where the independent variables were time (before and after) and the joint angles, and the dependent variables were the SWV and joint stiffness. When appropriate, post hoc comparisons were performed using the Bonferroni-corrected *t*-test. The level of significance was set at *P* < 0.05. We calculated the 95% confidence interval (CI) for the difference between the mean values. *partial η*^2^ was calculated as the effect size for the main effect and interaction in the two-way ANOVA where 0.01, 0.06, and 0.14 indicated small, medium, and high effects [23]. Additionally, the effect size (*r*) for the post hoc test was calculated [24]. Effect size *r* was classified as small (≤0.10 and <0.30), medium (≤0.30 and <0.50), and large (≤0.50) [25].

## 3. Results

**Figure 4:**
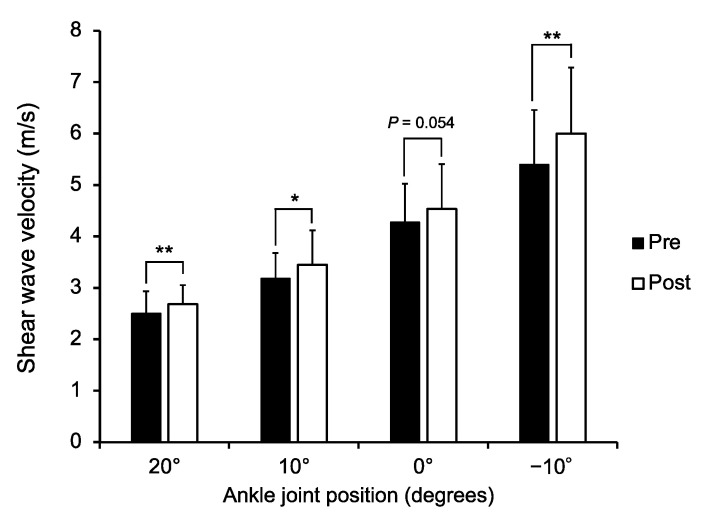
Shear wave velocity of the medial head of the gastrocnemius pre- and post-dermal suction for each ankle joint angle. * *P* < 0.05, ** *P* < 0.01.

No participant dropped out from the study during the entire study duration. The SWV of the MG for each joint angle before and after dermal suction is shown in Figure 4. The two-way repeated measures ANOVA revealed the significant main effects of time (*F* value = 11.682, *P* = 0.002, *partial η*^2^ = 0.337) and joint angle (*F* value = 287.860, *P* < 0.001, *partial η*^2^ = 0.926), and showed a significant interaction between the time and the joint angle (*F* value = 4.502, *P* = 0.006, *partial η*^2^ = 0.164). The post hoc comparison revealed that the SWV increased when the ankle joint was dorsiflexed (20° vs. 10°; *r* = 0.91, *P* < 0.001, 95% CI [−0.871, −0.578], 20° vs. 0°; *r* = 0.95, *P* < 0.001, 95% CI [−2.112, −1.508], 20° vs. −10°; *r* = 0.96, *P* < 0.001, 95% CI [−3.592, −2.620], 10° vs. 0°; *r* = 0.93, *P* < 0.001, 95% CI [−1.311, −0.860], 10° vs. −10°; *r* = 0.95, *P* < 0.001, 95% CI [−2.799, −1.964], 0° vs. −10°; *r* = 0.92, *P* < 0.001, 95% CI [−1.544, −1.048]). Post hoc comparison also showed that excluding the ankle position at 0°, SWV was higher after dermal suction than before dermal suction (20°; *r* = 0.53, *P* = 0.006, 95% CI [−0.301, −0.056], 10°; *r* = 0.43, *P* = 0.030, 95% CI [−0.496, −0.028], −10°; *r* = 0.60, *P* = 0.001, 95% CI [−0.941, −0.259]). A marginally significant difference was found between the pre- and post-dermal suction SWV values at 0° (*r* = 0.39, *P* = 0.054, 95% CI [−0.521, 0.004]).

**Figure 5:**
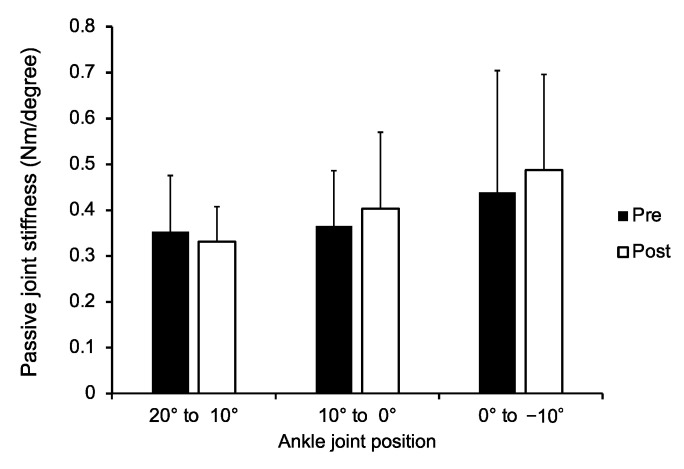
Passive joint stiffness of the ankle joint pre- and post-dermal suction for each ankle joint angle.

The passive ankle joint stiffness before and after dermal suction is demonstrated in Figure 5. The two-way repeated measures ANOVA revealed the significant main effects of the joint angle (*F* value = 10.091, *P* < 0.001, *partial η*^2^ = 0.305). In contrast, the main effect of time (*F* value = 2.362, *P* = 0.138, *partial η*^2^ = 0.093) and interaction between the time and the joint angle (*F* value = 2.207, *P* = 0.122, *partial η*^2^ = 0.088) were not significant. The post hoc comparison demonstrated that joint stiffness at 0° to −10° was higher than that at 20° to 10° (*r* = 0.55, *P* = 0.004, 95% CI [−0.206, −0.035]) and 10° to 0° (*r* = 0.46, *P* = 0.014, 95% CI [−0.144, −0.014]). There was no significant difference between the joint stiffness at 20° to 10° and 10° to 0° (*r* = 0.29, *P* = 0.225, 95% CI [−0.100, 0.016]). We carried out a post hoc test to calculate the power (1-*β*) by setting an *α* level of 0.05, the effect size for the main effects of time in SWV, and the sample size using G*Power (version 3.1.9.3). As a result, the power was 1.00.

## 4. Discussion

In the present study, we compared the passive muscle stiffness of the MG and passive ankle joint stiffness before and after dermal suction to verify the hypothesis that dermal suction decreases passive muscle stiffness and increases passive joint flexibility. Based on the results, two main findings were noted: (a) dermal suction significantly increased passive muscle stiffness; and (b) it had no significant effect on passive joint stiffness.

In contrast to our hypothesis, the current results showed a significant acute increase in the SWV after dermal suction, indicating that dermal suction acutely increases muscle stiffness. A possible explanation for the observed acute increase in muscle stiffness following dermal suction is that the suction may result in an increase in intramuscular water content. Yoshitake et al. [26] investigated the influence of the tissue covering the skeletal muscles on the mechanical properties of the muscles using shear wave elastography. The results demonstrated a 50% significant decrease in the muscle shear modulus after removal of the skin. This report [26] implied that the higher the internal pressure in the muscle, the greater the muscle stiffness. A recent study [27] examined the hemodynamic changes during cupping therapy and found that cupping therapy increased the blood volume and tissue oxygenation at the treatment site. Taking this evidence into consideration, it seems likely that dermal suction increases the blood flow and internal pressure in the muscle, which may increase muscle stiffness.

This phenomenon may manifest as an increase in the muscle volume after dermal suction. Thus, we additionally calculated the muscle thickness of the MG before and after dermal suction. The results showed no significant change in muscle thickness (cm) (20°: pre-dermal suction 1.64 [0.21] vs. post-dermal suction 1.68 [0.18], 10°: pre-dermal suction 1.71 [0.21] vs. post-dermal suction 1.77 [0.19], 0°: pre-dermal suction 1.78 [0.22] vs. post-dermal suction 1.80 [0.20], −10°: pre-dermal suction 1.83 [0.22] vs. post-dermal suction 1.80 [0.16], all *P* > 0.05). These findings, however, do not lead to the conclusion that the intramuscular water content did not affect our results. In the current study, muscle thickness was only measured in one slice. The changes in muscle thickness induced by resistance training have been reported to be different within the same muscle [28,29]. If there is an inhomogeneous change in the muscle thickness within the same muscle after the dermal suction, it is possible that the muscle thickness measured in only one slice may not have accurately indicated the acute increase in muscle volume due to dermal suction. Further studies attempting to clarify this point should be undertaken.

The results of the present study on muscle stiffness contradict those of a previous study, which found a decrease in muscle hardness after cupping [15]. Several factors might have affected the different outcomes of the two studies. As above-mentioned, several different ultrasound elastography methods are in use [11]: the previous study assessed muscle hardness using strain elastography [15], whereas in our study, muscle stiffness was assessed using shear wave elastography. Differences in the mechanical properties of the muscles measured might have led to the different results. In addition, the differences in target muscles, cup size, duration of treatment, and degree of vacuum pressure might be associated with the change in mechanical properties of the muscle after dermal suction.

The current results showed a significant increase in SWV, but not in passive joint stiffness (Figure 4 and Figure 5). Joint flexibility is influenced not only by the muscle but also by all the structures located around the joint including the tendon, skin, subcutaneous tissue, joint capsule, and cartilage [10]. Hence, it is possible that no significant changes were detected in passive joint stiffness because of the altered mechanical properties of the other tissues. Indeed, a previous animal study reported a significant decrease in skin stiffness after cupping therapy [30].

Moreover, the current finding regarding joint stiffness might be related to the intensity of dermal suction. Using the finite element method, a study revealed that stresses in the soft tissue increased with an increase in applied vacuum pressure during cupping therapy [2]. Hence, the application of suction at various intensities could result in a change in joint stiffness after dermal suction. As this point has not been considered in previous studies on cupping therapy and joint flexibility, the influence of the intensity of dermal suction on joint flexibility must be investigated.

Cupping and other dermal suction methods are widely used to reduce muscle stiffness or hardness [15] and to increase joint flexibility [5,6,7,8,9]. The results of this study showed a significant increase in muscle stiffness after applying dermal suction (Figure 4). A recent study revealed that higher passive muscle stiffness measured using shear wave elastography was significantly related to the superior sprint performance [31]. Furthermore, it has also been reported that stiffer muscles are advantageous with respect to the rapid force production (i.e., rate of torque development) [32]. From this viewpoint, an increase in muscle stiffness due to dermal suction may enhance sports performance. Conversely, we obtained no significant change in the joint stiffness among the participants in this study (Figure 5). Previous studies evaluating the effects of cupping therapy on the joint flexibility used joint ROM as an index of joint flexibility [5,6,7,8,9]. However, the joint ROM is influenced by the mechanical properties of tendons, muscles, ligaments, and joint capsules as well as psychological factors such as pain threshold, the subject’s desire to demonstrate progress in flexibility, and stretch tolerance [33,34]. Joint stiffness used in this study was an alternative approach that can eliminate the effect of psychological factors [33]. Physical therapists or conditioning professionals using dermal suction in their practice should understand the limited acute effects of dermal suction on joint stiffness.

The present study had some limitations. First, the intensity and duration of treatment by dermal suction were limited. We believe that multiple combinations of intensity and duration of treatment can provide useful data on dermal suction. Second, this study was not designed to quantify time-course changes (e.g., 10 min or 30 min after dermal suction). Therefore, it should be noted that this study only investigated the immediate effects of dermal suction. Third, we did not investigate the water intake of the subjects. We noted that intramuscular water content may be related to the increase in muscle stiffness after dermal suction in this study. Further experiments with controlled water intake conditions should be undertaken. Fourth, while the increase in muscle stiffness could be related to the increase in intramuscular water content, the actual mechanism underlying increased muscle stiffness is unclear. Further research including various methodologies such as biochemical or physiological analyses are needed to obtain detailed information on the effects of dermal suction on the mechanical properties of muscle.

## 5. Conclusions

The findings presented here provide information about the effects of dermal suction on the mechanical properties of muscle and joint. Our results suggest that dermal suction increases passive muscle stiffness and has a limited impact on passive joint stiffness. These results might indicate that dermal suction is useful when physical therapists or conditioning professionals are trying to increase muscle stiffness in their clinical practice. However, how muscles or joints are affected by dermal suction remains unclear. Well-constructed scientific research detailing the mechanism of action of dermal suction would help increase our understanding of this treatment.

## Figures and Tables

**Table 1:** Age and physical characteristics of the enrolled subjects.

	Sex	Mean	SD	95% CI lb	95% CI ub
Age (years)	Men	23.2	3.6	20.9	25.4
	Women	22.8	9.3	16.9	28.7
Height (cm)	Men	172.1	4.2	169.4	174.8
	Women	159.0	4.9	155.9	162.1
Body mass (kg)	Men	70.1	8.7	64.6	75.6
	Women	54.4	5.2	51.0	57.7
BMI (kg/m^2^)	Men	23.7	3.4	21.5	25.9
	Women	21.5	1.9	20.3	22.7

## Data Availability

The data of this study are available from the corresponding author, upon reasonable requests.
